# Placebo effects in low back pain: A systematic review and meta‐analysis of the literature

**DOI:** 10.1002/ejp.1811

**Published:** 2021-06-21

**Authors:** Johan (Hans) Peter Alexander van Lennep, Faye Trossèl, Roberto Silvio Giovanni Maria Perez, René Hubert Joseph Otten, Henriët van Middendorp, Andrea Walburga Maria Evers, Karolina Maria Szadek

**Affiliations:** ^1^ Department of Anesthesiology Amsterdam University Medical Center Amsterdam The Netherlands; ^2^ Health, Medical and Neuropsychology Unit Faculty of Social Sciences Leiden University Leiden The Netherlands; ^3^ Medical Library Vrije Universiteit Amsterdam Amsterdam The Netherlands; ^4^ Leiden Institute for Brain and Cognition Leiden The Netherlands; ^5^ Department of Psychiatry Leiden University Medical Center Leiden The Netherlands; ^6^ Medical Delta Leiden University, Technical University Delft, and Erasmus University Leiden The Netherlands

## Abstract

**Background and Objective:**

The current treatments of primary musculoskeletal low back pain (LBP) have a low to moderate efficacy, which might be improved by looking at the contribution of placebo effects. However, the size of true placebo effects in LBP is unknown. Therefore, a systematic review and meta‐analysis were executed of randomized controlled trials investigating placebo effects in LBP.

**Databases and Data Treatment:**

The study protocol was registered in the international prospective register of systematic reviews Prospero (CRD42019148745). A literature search (in PubMed, Embase, The Cochrane Library, CINAHL and PsycINFO) up to 2021 February 16th yielded 2,423 studies. Two independent reviewers assessed eligibility and risk of bias.

**Results:**

Eighteen studies were eligible for the systematic review and 5 for the meta‐analysis. Fourteen of the 18 studies were clinical treatment studies, and 4 were experimental studies specifically assessing placebo effects. The clinical treatment studies provided varying evidence for placebo effects in chronic LBP but insufficient evidence for acute and subacute LBP. Most experimental studies investigating chronic LBP revealed significant placebo effects. The meta‐analysis of 5 treatment studies investigating chronic LBP depicted a significant moderate effect size of placebo for pain intensity (*SMD* = 0.57) and disability (*SMD* = 0.52).

**Conclusions:**

This review shows a significant contribution of placebo effects to chronic LBP symptom relief in clinical and experimental conditions. The meta‐analysis revealed that placebo effects can influence chronic LBP intensity and disability. However, additional studies are required for more supporting evidence and evidence for placebo effects in acute or subacute LBP.

**Significance:**

This systematic review and meta‐analysis provides evidence of true placebo effects in low back pain (LBP). It shows a significant contribution of placebo effects to chronic LBP symptom relief. The results highlight the importance of patient‐ and context‐related factors in fostering treatment effects in this patient group. New studies could provide insight into the potential value of actively making use of placebo effects in clinical practice.

## 1 INTRODUCTION

Primary musculoskeletal low back pain (LBP) is defined as pain limited to the region between the lower margins of the 12th rib and the gluteal folds, with or without leg pain (Anderson, 1977; Chou, 2010; Treede et al., 2019; Van Tulder et al., 2006). The symptoms can be divided in acute (persisting <6 weeks), subacute (persisting 7–12 weeks), or chronic (persisting >12 weeks) and are often treated accordingly (Oliveira et al., 2018). Treatment for LBP consists of a broad spectrum of pharmacological and nonpharmacological interventions, for example nonsteroidal anti‐inflammatory drugs (NSAIDs), muscle relaxants, and multidisciplinary rehabilitation (Chou et al., 2016; Oliveira et al., 2018). However, most of these treatments have a low to moderate effect on pain and disability when compared to sham interventions (Chou et al., 2016; Machado et al., 2009). One explanation for this might be a substantial placebo effect seen in sham interventions (Testa & Rossettini, 2016). The placebo effect is the symptomatic improvement in any condition due to the psychosocial context surrounding a sham or true medical intervention (Benedetti, 2013; Evers et al., 2018). In the case of pain symptoms, it is able to produce an analgesic response comparable to that of remifentanil (Atlas et al., 2012). Therefore, inducing placebo effects during medical treatments, for instance by paying attention to contextual factors, might help clinicians increase LBP treatment efficacy (Blasini et al., 2018; Testa & Rossettini, 2016).

In a previous systematic review, the effect of placebo treatments in LBP was studied as change in pain scores from baseline (Puhl et al., 2011). The authors performed a best‐evidence synthesis and discovered a clinical meaningful change in pain scores ranging from 41% to 55% of study subjects after different placebo treatments. Although these results show a meaningful influence of treatment context, they fail to discriminate between placebo effects and nonspecific effects. Nonspecific effects are all treatment effects seen in sham intervention groups, which can be attributed not only to placebo effects but also to other contextual factors such as the natural history of the disease, regression to the mean, and experimenter biases (Benedetti et al., 2003). One way of distinguishing placebo effects from nonspecific effects is to investigate trials with an additional no‐treatment group that also entails all nonspecific components except for the placebo effect (Klinger et al., 2018). Comparing the improvement of the placebo group with the no‐treatment group subsequently reveals the true size of the placebo effect. In this systematic review, we therefore aim to investigate the size of the placebo effect in LBP by comparing placebo (or sham) and no‐treatment control groups, including a meta‐analysis, and consecutively explore the role of different sham interventions in LBP. Executing a meta‐analysis could reveal the size of the placebo effect and subsequently inform treatment providers to what extent stand‐alone placebo interventions or contextual factors might improve LBP treatment (Mbizvo et al., 2015).

## 2 LITERATURE SEARCH METHODS

### 2.1 Protocol and registration

This study was conducted in accordance with the PRISMA guidelines (Moher et al., 2009) and following the Cochrane Handbook for systematic reviews of interventions (5.1.0) (Higgins & Green, 2011). The study protocol was registered in the international prospective register of systematic reviews Prospero (CRD42019148745).

### 2.2 Eligibility criteria

#### 2.2.1 Types of studies

Clinical treatment randomized controlled trials (RCTs) with no‐treatment and placebo arms or experimental RCTs were considered for inclusion. Both parallel and cross‐over designs were eligible for inclusion. Only English trials or English‐translated trials in humans were studied.

#### 2.2.2 Types of participants

Patients aged 18–75, with primary musculoskeletal LBP that met the following criteria: pain below the 12th costal margin and above the inferior gluteal folds, without radiation below the knee, for which other etiologies such as infection, tumor, osteoporosis, fracture, structural deformity, inflammatory disorder, radicular syndrome or cauda equina syndrome, and other relevant pathological entities had been excluded (Puhl et al., 2011). Patients with any duration of LBP were included: acute, subacute, and chronic. Placebo effects were studied per duration category because (1) acute and subacute LBP often have a self‐limiting course, (2) LBP patients are often treated according to study duration, and (3) there is evidence for an altered response to placebo effects in patients with chronic pain symptoms (Kaptchuk et al., 2020; Oliveira et al., 2018). Studies that did not specifically assess patients according to LBP symptom duration were not included in the meta‐analysis part of this review since analysing placebo effects according to symptom duration would not be possible.

#### 2.2.3 Types of interventions

Placebo intervention was defined as any intervention specified as placebo in the investigation record. Equivalent terms for placebo were sham, dummy, counterfeit, or fake. Also, to prevent exclusion from trials not describing their placebo intervention but mentioning placebo effects, a specific placebo effect term was integrated in the search. Equivalent terms for this were placebo response or placebo reaction.

A no‐treatment control group consisted of patients not receiving an experimental or placebo treatment. Other terms for this no‐treatment group were nontreatment and no‐drug intervention or therapy. Trials investigating a no‐treatment group described as a usual care group were included in the review when compared to a group that investigated placebo interventions plus usual care.

#### 2.2.4 Types of outcome measures

Primary outcomes were changes in pain intensity on any scale (e.g., Visual Analogue Scale (VAS) (Price et al., 1983), Numeric Rating Scale (NRS), Brief Pain Inventory (BPI) (Cleeland & Ryan, 1994) intensity questions, Short form McGill Pain Questionnaire (SF‐MPQ) (Smith et al., 2015)) or changes in LBP‐related disability (e.g., Roland Morris Disability Questionnaire (RMQ) (Roland & Morris, 1983), Oswestry Disability Index (ODI) (Fairbank et al., 1980) and Quebec Pain Disability Scale (Kopec et al., 1995)).

#### 2.2.5 Search methods for identification of studies

A literature search was performed by R.O. and H.v.L. To identify all relevant publications about the placebo effect with low back pain, we performed systematic searches in the bibliographic databases PubMed, EMBASE, The Cochrane Library (via Wiley), CINAHL and PsycINFO (both via EBSCO) from inception to 2021 February 16th. Search terms included controlled terms (e.g., MeSH in PubMed and Emtree in Embase) as well as free text terms. Free text terms were used only in The Cochrane Library. Search terms expressing ‘back pain’ were used in combination with search terms comprising the placebo effect. All languages were accepted. The references of the identified articles were searched for relevant publications. The full search strategies for all databases can be found in Appendix S1. After conducting the search, two review authors (H.v.L. and F.T.) selected titles and abstracts that possibly met the eligibility criteria. The full texts of these articles were then obtained and again assessed by both authors for final eligibility. Any doubts were resolved through discussion with a third author (K.S.).

### 2.3 Data extraction

The first author (H.v.L.) extracted the following data from each article: (1) mean and standard deviation (*SD*) values of pain rating scales or medians and interquartile ranges (IQR) in the case of nonnormally dispersed data and (2) demographic, clinical and placebo characteristics (e.g., number of patients, age, gender, duration of LBP, applied placebo intervention, study design, sample sizes in placebo and no‐treatment groups, duration of placebo treatment, type of analysis, publication status). Unreported means and standard deviations in studies were either constructed statistically based on the information as proposed by the *Cochrane Handbook for Systematic Reviews of Interventions* (Higgins & Green, 2011) or obtained by contacting the corresponding author.

### 2.4 Risk of bias assessment

Risk of Bias (RoB) was systematically assessed by two independent reviewers (H.v.L. and F.T.) according to the tool of the *Cochrane Handbook for Systematic Reviews of Interventions* (Higgins & Green, 2011). The following types of bias were judged: (1) selection bias—description and interpretation of random sequence generation and concealed allocation; (2) performance bias—blinding of participants, and personnel; (3) detection bias—blinding of outcome assessors; (4) attrition bias—comprehensiveness of the reported outcome data; (5) reporting bias—the selective reporting of outcomes; and (6) other sources of bias. For other bias, two different aspects were evaluated: more than two intervention groups and possible carry‐over effects in crossover studies. Discrepancies between the reviewers were resolved by discussion with a third author (K.S.).

### 2.5 Sensitivity analysis

Primarily, box plots were used in the exploratory phase of the data analysis to assess the dispersion of the data. Hereafter, random effects models were investigated by the leave‐one‐out method for sensitivity analysis. Presence of publication bias was assessed by visually inspecting funnel plots (Greenhouse & Iyengar, 1994). If the number of included studies deemed insufficient for visual interpretation, Egger's test was used as a more extensive approach (Egger et al., 1997). In this linear regression analysis method, the effect sizes of the individual studies are plotted against a precision measure (the inverse of the standard error) and in this plot deviations of the intercept from zero are considered a sign of publication bias. To correct for possible publication bias in the meta‐analysis, funnel plots were examined according to the ‘trim and fill method’.

### 2.6 Statistical analysis

For statistical analyses, IBM SPSS software (version 26) was used. Meta‐analyses were performed with Revman Analyses software (version 5.3) of the Cochrane Collaboration. Effect sizes for the individual trials were calculated with the standardized mean difference (*SMD*) in RevMan for the pain and/or disability scales. The effect sizes were obtained by subtracting means and standard deviations of placebo groups from no‐treatment groups. The meta‐analysis was conducted with a random effects model, which was preferred over a fixed effects model due to the expected heterogeneity (DerSimonian & Laird, 2015). To obtain the ‘weight’ for estimating the overall population treatment effect (μ) and the contributing standard error, a variation of the inverse‐variance methods by DerSimonian and Kacker (2007) was used. Furthermore, confidence intervals, standard errors and Z‐scores were computed from the overall population treatment effect (µ), that is, summary effect size (Shadish & Haddock, 1994). A two‐sided value of *p* ≤ .05 was considered significant in all analyses. The impact of heterogeneity on the meta‐analysis was assessed by conducting the *I^2^* test and visually inspecting forest plots. Values for the *I^2^* test of 50%–90% were roughly interpreted as substantial heterogeneity across studies. To illustrate the amount of heterogeneity by subgroup differences, *τ^2^* was calculated (Higgins & Green, 2011).

## 3 RESULTS

### 3.1 Search results

**FIGURE 1:**
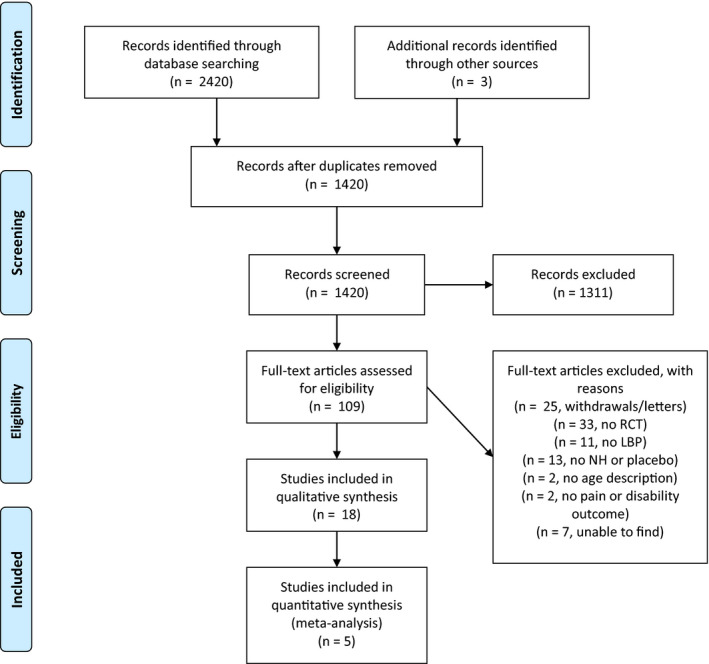
PRISMA flow diagram, search up until 2021, showing study selection process, including reasons for exclusion. Selection was conducted by 2 reviewers

The search up until 2021 resulted in identification of 2,420 records through electronic databases and 3 more records through reference lists, yielding a total of 2,423 records. After removing 1,003 duplicates, 1,420 records were screened for eligibility. Careful inspection of the titles and abstracts resulted in exclusion of 1,311 records not meeting the inclusion and exclusion criteria. For the resulting records, full texts were reviewed and after exclusion of another 91 articles, a total of 18 articles were included in the systematic review (Figure 1).

Close inspection of the 18 included articles of both searches yielded 5 studies that were eligible for a meta‐analysis. Authors were contacted to provide missing data essential for pooling the results of those studies.

### 3.2 Systematic review

The characteristics and outcomes of the studies included in the systematic review are described in Table 1.

**TABLE 1:** Characteristics of included studies

Reference	LBP type, duration.	Total *N* in placebo and control groups, mean age (*SD*) and percentages females	Study design	Outcome measurement	Placebo group	Control group	Protocol	Results
Bialosky et al., (2014)	Not specified, Duration: 16 weeks	*N* = 72, Age: 31.68 (11.85) Females (%) : 64%	RCT	0 (baseline) and 2 weeks (postintervention)	Sham manipulative therapy and enhanced sham by instructions	No‐treatment: sit down and wait.	5 therapy sessions in 2 weeks, each lasting 5 min.	*Clinical pain* (intensity)*:* Baseline: NRS placebo; 43.78 (22.45)/NRS control; 33.93 (26.21) Within groups: NRS placebo; 29.88 (20.64), DS: 14.04 (3.51)/NRS control; 25.73 (22.04), DS: 8.20 (2.39) Between (all) groups: *p* =.68, ES (η^2^) = 0.02 *Disability:* Baseline: ODI placebo; 14.22 (8.56)/ODI control; 20.04 (15.27) Within groups: ODI placebo: 12.23 (10.22), DS: 2.08 (1.44)/ODI control: 17.50 (12.66), DS: 2.53 (1.60) Between (all) groups: *p* =.73, ES (η^2^) = 0.01
Borges et al. (2013)	CLBP, Duration: 4.4 years	*N* = 29 Age: 39.6 (9.6) Females (%) : 77%	RCT	0 (baseline), 3 and 6 weeks (postintervention)	Sham laser application on acupressure points	No‐intervention	12 therapy sessions in 6 weeks, each lasting 20 min.	*Clinical pain* (intensity)*:* Baseline: NRS placebo; 5.7 (1.0)/NRS control: 5.0 (1.2) Within groups: NRS placebo; 4.8 (1.5)–4.7 (1.0), *p* >.05, ES(d) = 17% /NRS control; 5.3 (1.0)–5.9 (1.2), *p* >.05, ES(d) = −17% Between groups ^a^: Z = 1.06, *p* =.29, ES(smd) = 0.38 *Disability:* not assessed
Brinkhaus et al., (2006)	CLBP, Duration: 14.7 (11.1) years	*N* = 144 Age: 58.8 (9.1) Females (%) : 68%	RCT	0 (baseline), and 8 weeks.	Minimal acupuncture with needling of nonspecific areas.	Waiting list group could use NSAIDs as back up medication	12 sessions of 30 min in 8 weeks; in the first 4 weeks twice a week, than weekly for remaining 4 weeks.	*Clinical pain* (intensity): Baseline: NRS placebo; 66.6 (15.7)/NRS control; 66.1 (13.6) Within groups: NRS placebo; 43.7 (29.8), DS 23.6 (31.0)/NRS control; 58.6 (25.1), DS 6.9 (22.0) Between groups ^a^: Z = 3.18, *p* =.001, ES(smd) = 0.54 *Disability:* Baseline: PDI placebo; 31.5 (11.1)/PDI control; 31.0 (13.3) Within groups: PDI placebo; 21.5 (13.2)/PDI control; 27.1 (14.1). Between groups ^a^: Z = 4.02, *p* <.001, ES(smd) = 0.69
Bush et al., (1985)	CLBP, Duration: 10–13 years	*N* = 48, Age: 20–65 yrs. Females (%): 47%	RCT	0 (baseline), postintervention and 3 months (follow‐up)	Stabilization of back with temperature feedback	Waiting list group	At least 8 intervention sessions, each lasting 40 min. + exercises at home 4/day.	*Clinical pain* (intensity)*:* Baseline: NRS placebo; 3.37/NRS control; 3.32 Within groups: not shown Between (all) groups: MANOVA for DLBP and MGPQ was not significant *Disability:* not assessed
Carvalho et al., (2016)	CLBP, Duration: not reported	*N* = 76, Age: Placebo: 44.4 (13.2) Control: 44.1 (13.7) Females (%) : 71%	RCT	0 (baseline), and 3 weeks.	Open label placebo where participants are informed that they will receive an inert substance.	Usual care, after 3 weeks of testing participants could still use placebo pills if they wanted to.	Placebo pills twice a day for 3 weeks.	*Clinical pain* (intensity): Baseline: NRS placebo; 4.8 (1.8)/NRS control; 5.0 (1.7) Within groups: NRS placebo; DS 1.48 (1.79)/NRS control; DS 0.44 (2.13) Between groups: *F* = 11.02, *p* <.001, ES(g) = 0.76 *Clinical pain* (bothersomeness): Baseline: NRS placebo; 6.0 (2.1)/NRS control; 5.6 (2.3) Within groups: NRS placebo; 1.44 (2.46)/NRS control; 0.78 (2.61) Between groups: *F* = 1.71; *p* =.195 ES(g) = 0.21 *Disability:* Baseline: RMDQ placebo; 8.5 (4.6), RMDQ control; 9.9 (5.2) Within groups: RMDQ placebo; DS 2.86 (3.91)/RMDQ control; DS 0.02 (3.73) Between groups: *F* = 12.10, *p* <.001, ES(g) = 0.74
Charron et al., (2006)	CLPB, Duration: 8.4 (6.9) years	*N* = 16, Age: 39.8 (13.2) Females (%): 37.5%	Cross‐over study, pseudo random.	0 (baseline) and postintervention	Injection of 1ml saline with a specific instruction	Injection of 1ml saline with a control instruction	Two sessions on different days, lasting for 3 hr, receiving both placebo or control on different days.	*Clinical pain* (intensity): Baseline: not reported Within groups: MANOVA placebo; *p* =.08/MANOVA control; *p* >.05. Between groups: *p* =.142 *Clinical pain*(unpleasantness): Baseline: not reported Within groups: MANOVA placebo; *p* =.038/MANOVA control; *p* >.05 Between groups: *p* =.046 *Disability:* not assessed
Cherkin et al., (2009)	CLBP, Duration: ≥3 months	*N* = 363 Age: 47 (13) Females (%): 62%	RCT	0 (baseline), 8, 26 and 52 weeks.	Simulated acupuncture with a toothpick and specific instructions, no skin penetration.	Usual care consisting of primary care, pain medications and physiotherapy visits.	10 treatment sessions lasting 20 min in 7 weeks, in the first 3 weeks twice a week, than weekly for remaining 4 weeks.	*Clinical pain* (bothersomeness): Baseline: NRS placebo; 4.9 (2.3)/NRS control; 5.4 (2.3) Within groups: NRS Placebo; 3.0 (2.4), 3.5 (2.7), 3.4 (2.7)/NRS control; 4.7 (2.6), 4.4 (2.6), 4.1 (2.6) Between groups: (placebo >control) 8 weeks MD −1.56 (95% CI −2.11 to −1.02) *p* <.05 26 weeks MD −0.78 (95% CI −1.36 to −0.19) *p* <.05 52 weeks MD −0.62 (95% CI −1.21 to −0.03) *p* <.05 *Disability:* Baseline: RMDQ placebo; 9.8 (5.1)/RMDQ control; 11.0 (5.1) Within groups: RMDQ placebo; 5.4 (4.9), 6.4 (6.0), 6.2 (5.8)/RMDQ control; 8.9 (6.0), 8.4 (6.0), 7.9 (6.5) Between groups: (placebo >control) 8 weeks MD −2.91 (95% CI −3.96 to −1.86) *p* <.05 26 weeks MD −0.78 (95% CI −1.36 to −0.19) *p* <.05 52 weeks MD −0.98 (95% CI −2.11 to 0.14) *p* = NS
Degenhardt et al., (2014)	CLPB, Duration: 9.1 (6.3) years	*N* = 20, Age: 36 (11) Females (%) : 73%	RCT	0 (baseline), 1 and 24 hr postintervention	Sham ultrasound	No‐intervention	A single session of 60 min, sham ultrasound lasted 20 min and afterwards 40 min resting.	*Clinical pain* (intensity): Baseline ^b^: NRS placebo; 4 (3;4)/NRS control; 4 (2;6) Within groups ^b^: NRS placebo; 3(1:3), 2.5(2:3), Friedman: *p* =.01/NRS control; 3.5(2:6), 3.5(2:5), Friedman: *p* =.64 Between (all) groups: nonparametric testing at 1h and 24 hr; *p* =.37, *p* =.51 *Disability:* not assessed
Eardley et al., (2013)	CLPB, Duration: (per group) SPKP: 11.6 (8.6) WLC: 9.2 (6.6)	*N* = 46, Age: Sham PKP: 48.1 (10.6) Waiting list control: 44.6 (10.3) Females (%) : 60%	RCT	0 (baseline), after every week in first 5 weeks (pain), 5 weeks (postintervention) and 7 weeks (follow‐up).	Sham PKP with sham rechecks and without verbal advice	Waiting list group for 6 weeks, than randomized to sham or PKP	5 therapy sessions in 5 weeks, each lasting 4–60 min.	*Clinical pain* (intensity): Baseline: VAS placebo; 51.8 (19.2)/VAS control; 52.2 (20.5) Within groups: VAS placebo; 36.0 (19.8), DS −15.8(18.1)/VAS control; 57.7 (19.2), DS 3.8 (20.9). Between groups: MD −11.9 (95% CI −20.8; −23.0), *p* =.10 *Disability:* Baseline: RMDQ placebo; 11.3 (4.1)/RMDQ control; 10.4 (5.2) Within groups: RMDQ placebo; 4.9(4.5), DS −6.4(5.2)/RMDQ control; 10.1(5.2), DS 0.0(4.6). Between groups: MD −6.1 (95% CI −5.9; 0.1), *p* <.005, ES = 1.4
Faas et al., (1993)	ALBP, Duration: ≤ 3 weeks	*N* = 280, Age: 36 Females (%) : 43%	RCT	0 (baseline), 1 month, 3 months and 12 months (follow‐up)	Sham ultrasonography with lowest possible dose next to zero (0.1 watt/cm^2^)	Treatment as usual(painkillers and instructions)	10 sessions in 5 weeks (twice a week) lasting 20 min.	*Clinical pain* (intensity): Baseline: NRS placebo; 36.6/NRS control; 38.1 Within groups: NRS placebo; DS −19 (23), −22 (28), −26 (26)/NRS Control; DS −19 (21), −24 (24), −26 (23). Between groups ^a^: Z = 0.00, *p* = 1.00, ES(smd) = 0.00 *Disability:* Baseline: NRS placebo; 23.3/NRS control; 24.3 Within groups: NRS placebo; DS −10 (18), −13 (19), −15 (19)/NRS Control; DS −9 (19), −12 (20), −14 (19). Between groups ^a^: Z = 0.45, *p* =.65, ES(smd) = −0.05.
Ikemoto et al., (2020)	CLBP, Duration ≥3 months	*N* = 52, Age: 66.8 (13.4) Open label placebo: 68.2 (13.0) Treatment as usual: 65.3 (13.8) Females (%): 68.2%	RCT	0 (baseline), week 3 (during intervention), and week 12 (post‐intervention)	Open label placebo where participants are informed that they will receive an inert substance.	Treatment as usual (education and painkillers)	Placebo pills twice a day for 12 weeks	*Clinical pain* (intensity): Baseline: NRS placebo; 5.3 (1.9)/NRS control; 5.5 (1.6) Within groups: NRS placebo; DS −0.9 (1.8), −1.1 (1.9)/NRS control; −0.2 (1.8), −0.8 (1.9) Between groups: placebo >control at 3 weeks *p* =.19, *d* = 0.38/ at 12 weeks placebo >control *F* = 1.00, *p* =.37, η2 = 0.05 *Disability:* Baseline: RMDQ placebo; 9.9 (3.7)/RMDQ control; 10.3 (4.0) Within groups: RMDQ placebo; −2.2 (2.9)/RMDQ control; −1.4 (3.6) Between groups: at 3 weeks placebo >control; *p* =.40; *d* = 0.24 at 12 weeks placebo >control *F* = 0.82, *p* =.37, η2 = 0.02
Kim et al., (2020)	CLBP ≥6 months. Duration: (per group) SA: 10.6 years (10.8) ML: 6.0 years (5.4) TAU: 9.2 years (7.6)	*N* = 60 Age: 41.2 (12.0) Sham acupuncture: 41.8 (12.2) Mock laser acupuncture: 41.7 (12.3) Treatment as usual: 39.1 (9.8) Females (%): 52%	RCT	0 (baseline) and during 4 weeks treatment period	Sham acupuncture: acupoints were pressured with non‐penetrating Streitberger needles Mock laser acupuncture: laser was held over acupoints for 15 s.	Treatment as usual (education +painkillers)	Six sham or mock acupuncture treatments over 4 weeks: twice per week in the first half and once per week in the second half.	*Clinical pain* (bothersomeness): Baseline: not reported Within groups: not reported Between (all) groups: *F* = 0.26, *p* =.86 *Disability:* not assessed
Klinger et al., (2017)	CLBP, Duration: (per group) OI: 119.33 (75.23) months OI +cond: 158.83 (138.96) months PI: 165.67 (117.17) months PI +cond: 162.17 (106.86) months	*N* = 48, Age: 49.97 (13.64) Females (%): 75%	RCT	0 (baseline), before and after first exercise, before and after first solution (postintervention), before and after second exercise, before and after second solution (postintervention).	Opioid instruction (with or without conditioning) about an oral saline solution	Open placebo instruction (with or without conditioning) about an oral saline solution	One session of 2 hr during which 2 solutions were administered after exercises. Conditioning happened between the administration of solutions.	*Clinical pain*(intensity): Baseline: VAS Placebo; 5.00 (2.09)/VAS Placebo +cond; 5.08(1.73)/VAS control; 4.67(1.67)/VAS control +cond; 5.25(1.87) Within groups: VAS Placebo; 4.00 (2.13), 3.00(2.73), *p* <.01, *d* = 0.83/VAS Placebo +cond; 3.58(1.56), 1.92(1.73), *p* <.01 *d* = 1.83/VAS control; 5.00(1.76), 5.58(1.78), *p* <.01, *d* = −0.64/VAS control +cond; 5.25(1.55), 4.58(2.31), *p* <.26, ES(d) = 0.32. Between groups: VAS placebo +cond > placebo; MD: −1.08 p: 1.00/VAS placebo +cond > control +cond; MD: −2.67 p: 0.03/VAS placebo +cond > control; MD: −3.92 p: 0.001/VAS placebo >control + cond; MD: −1.58 *p*: 0.52/VAS placebo >control; MD: −2.83 *p*: 0.018/VAS control +cond > control; MD: −1.25 *p* = 1.00 *Disability:* Baseline: FC placebo; 52.52(22.89)/FC placebo +cond; 60.56(20.78)/FC control; 51.11(15.13)/VAS control +cond; 50.00(20.99) Within groups: FC placebo; 67.78(29.24), *p* <.01, ES(d) = −0.59/FC placebo +cond; 77.22(15.43), *p* <.01, *d* = −0.92/FC control; 44.44(15.66), *p* =.06, ES(d) = 0.43/FC control +cond; 53.89(24.03), *p* =.22 ES(d) = −0.17 Between groups ^a^: FC placebo +cond > FC control +cond; Z = 2.51, *p* =.01, ES(smd) = 1.12/FC Placebo >FC control; Z = 2.21, *p* =.02, ES(smd) = 0.97
Leibing et al. (2002)	CLPB, Duration: 9.6 (8.2) years	*N* = 91, Age: 48.1 (9.7) Females (%): 58%	RCT	0 (baseline), 12 weeks (postintervention) and 9 months (follow‐up)	Active physiotherapy for 12 weeks (26 sessions) followed by minimal acupuncture for 12 weeks(20 sessions)	Active physiotherapy for 12 weeks, during testing treatment with painkillers	20 intervention sessions in 12 weeks, each lasting 20 min.	*Clinical pain* (intensity): Baseline: VAS placebo; 5.3 (1.8)/VAS control; 5.4 (1.9) Within groups: VAS placebo; 3.2 (1.9)–3.4 (2.1), DS −2.1 (2.2), −1.8 (2.2)/NH; 4.4 (2.5)–4.5 (2.7), DS −1.0 (1.7), −0.9 (2.0) Between groups ^a^: Z = 2.52, *p* =.01, ES(smd) = 0.54 *Disability*: Baseline: PDI placebo; 25.5 (10.4)/PDI control; 24.9 (13.7) Within groups: PDI placebo; 15.7 (10.1)–16.9 (11.6), DS −9.7 (10.5) −8.5 (11.3) /PDI control; 22.2 (15.4)–22.6 (15.8), DS −2.6 (7.8), −2.3 (10.0) Between groups ^a^: Z = 2.32, *p* =.02, ES(smd) = 0.49
Pires et al. (2020)	CLBP ≥3 months, Duration: (per group) Placebo: 44.4 (52.6) months Control: 58.2 (76.2) months	*N* = 42, Age: Placebo: 38.9 (10.3) Control: 45.3 (12.0) Females (%): 75%	RCT	0 (baseline), post‐intervention, and at 30 min (follow‐up).	Missner® surgical tape (5 cm in width) placed on the longissimus muscle.	No bandage	One session during which tape was applied after exercise intervention.	*Clinical pain* (intensity): Baseline: NRS placebo; 7.2 (1.3)/NRS control; 7.0 (2.5) Within groups: NRS placebo; 6.0 (2.8)–5.0 (3.2)/NRS control; 5.0 (3.2)–5.0 (3.2) Between groups: Post‐intervention MD: 0.24 (95% CI −1.06; 1.54), *p* =.72 At 30 min MD: −0.52 (95% CI −1.83; 0.78) *Disability:* Baseline: RMDQ placebo; 13.2 (5.3)/RMDQ control; 12.5 (6.7) Within groups: not assessed Between groups: not assessed
Pach et al., (2011)	CLBP, Duration: 15 (12) years	*N* = 87, Age: 57 (11) Females (%): 64%	RCT	0 (baseline), 8 and 26 weeks.	Placebo group received an injection with isotonic saline (rescue pain medication)	Rescue pain medication with peripherally acting analgesics	12 treatment sessions in 8 weeks; twice per week in the first half, than once per week in the second half.	*Clinical pain* (intensity): Baseline: VAS placebo; 62.5 (13.9)/VAS control; 59.0 (14.1) Within groups(mean and 95% CI): VAS placebo; 41.8 (30.1; 53.6), 35.5 (24.2; 46.9)/VAS control; 53.0 (41.8; 64.2), 45.0 (34.1; 55.9). Between groups ^a^: Z = 1.42, *p* =.15, ES(smd) = 0.31 *Disability:* Baseline: PDI placebo; 29.0 (13.8)/PDI control; 27.7 (11.8), HFAQ placebo; 61.3 (21.8)/HFAQ control; 65.5 (17.4) Within groups(mean and 95% CI): PDI placebo; 24.1 (17.7–25.1), 25.9 (22.5; 29.3)/PDI control: 25.9 (22.5; 29.3), 22.7 (18.7; 26.7). HFAQ placebo; 68.4 (63.8; 73.0), 67.4 (61.0; 73.8)/HFAQ control; 64.8 (60.5; 69.1), 64.8 (58.8; 70.9). Between groups ^a^: Z = 1.54, *p* =.12, ES(smd) = 0.33
Sanders et al., (1990)	ALBP, Duration: <2 weeks	*N* = 12, Age: Males: 41 (30.9) Females 33 (8.6) Females (%): 50%	RCT	0 (baseline), 5 and 30 min (postintervention)	Sham manipulation	No intervention	A single intervention, duration not specified.	*Clinical pain* (intensity): Baseline: not reported Within groups: not reported Between groups: no significant difference. *Disability*: not assessed
Vas et al., (2012)	ALBP, Duration: (per group) Sham: 5.0 (3.6) days Placebo: 6.5 (4.1) days Control: 6.8 (3.9) days	*N* = 207, Age: Sham: 44.0 (9.4) Placebo: 43.6 (12.2) Control: 41.2 (12.0) Females (%): 58%	RCT	0 (baseline), 3, 12 and 48 weeks (follow‐up).	Sham acupuncture: needling nonspecific points, Placebo acupuncture: temporarily applied pressure with a blunt needle.	Conventional Therapy (painkillers and instructions)	5 sessions in 2 weeks lasting 20 min.	*Clinical pain* (intensity): Baseline: VAS sham; 71.5 (18.3)/VAS placebo; 68.3 (19.5)/VAS control; 69.5 (18.0) Within groups: not reported Between groups: not reported *Disability:* Baseline: RMDQ Sham; 12.6 (6.3)/RMDQ Placebo; 14.1 (5.2)/RMDQ control 12.4 (5.1) Within groups: RMDQ sham; DS 65.0 (40.5), 84.7 (28.9), 75.6 (37.4)/RMDQ placebo; DS 43.1 (58.9), 76.3 (41.5), 70.7 (55.4)/RMDQ control; DS 26.6 (58.9), 83.0 (23.2), 63.3 (57.4). Between groups ^a^: Z = 3.79, *p* <.001, ES(smd) = 0.75

Abbreviations: ALBP, acute low back pain; CLBP, chronic low back pain; DLBP, Daily Low Back Pain Scale; DS, Difference Score; HFAQ, Hannover Functional Ability Questionnaire; MGPQ, McGill Pain Questionnaire; ML, Mock Laser Acupuncture; NRS, Numeric Rating; NSAID, NonSteroidal Anti‐Inflammatory Drugs; OI, Opioid Instruction; PDI, Pain Disability Index; PI, Placebo Instruction; PKP, Professional Kinesiology Practice; RCT, Randomized Controlled Trial; RMDQ, Roland Morris Disability Questionnaire; SA, Sham Acupuncture; SPKP, Sham Professional Kinesiology Practice; TAU, Treatment As Usual; WLC, Waiting List Control.

^a^ Between group analyses are conducted by the reviewers.

^b^ Numbers are reported as median and interquartile ranges.

#### 3.2.1 Population

All 18 studies included participants that were middle aged, with means ranging from 32 to 67 years (Table 1). In most studies (k = 14), more females were included than males. Fourteen clinical studies aimed at investigating patients with chronic LBP with averages in duration of pain ranging from 4.4 to 15.0 years (Borges et al., 2014; Brinkhaus et al., 2006; Bush et al., 1985; Carvalho et al., 2016; Charron et al., 2006; Cherkin et al., 2009; Degenhardt et al., 2014; Eardley et al., 2013; Ikemoto et al., 2020; Kim et al., 2020; Klinger et al., 2017; Leibing et al., 2002; Pach et al., 2011; Pires et al., 2020). Of these, 10 were clinical treatment studies comparing placebo interventions to a no‐treatment control (Borges et al., 2014; Brinkhaus et al., 2006; Bush et al., 1985; Cherkin et al., 2009; Degenhardt et al., 2014; Eardley et al., 2013; Kim et al., 2020; Leibing et al., 2002; Pach et al., 2011; Pires et al., 2020) and 4 were experimental studies specifically assessing placebo effects (Carvalho et al., 2016; Charron et al., 2006; Ikemoto et al., 2020; Klinger et al., 2017). Three clinical treatment studies investigated patients with acute LBP with a duration of less than 3 weeks (Faas et al., 1993; Sanders et al., 1990; Vas et al., 2012), and one study explored patients with subacute and chronic LBP (Bialosky et al., 2014).

#### 3.2.2 Placebo interventions

##### Sham acupuncture

The most frequent placebo intervention was sham acupuncture, utilized in 5 out of 18 studies. Two studies utilized minimal acupuncture to mimic real acupuncture, which meant that needles were only placed superficially, distant from the real acupuncture points and typical acupuncture stimulation was not given (Brinkhaus et al., 2006; Leibing et al., 2002; Vas et al., 2012). One of these studies also implemented a second placebo acupuncture group (simulated acupuncture) where acupuncture was mimicked by applying pressure with blunt needles (Vas et al., 2012). A similar procedure was executed in the remaining 2 studies where toothpicks or Streitberger needles that gave a painful sensation imitated the acupuncture (Cherkin et al., 2009; Kim et al., 2020). A mock laser that was held over acupoints was used in 1 of these studies to serve as a second sham group (Kim et al., 2020).

##### Sham manipulation

Sham manipulation was used in 3 out of 18 studies to imitate clinical interventions (Bialosky et al., 2014; Eardley et al., 2013; Sanders et al., 1990). In one study, a sham manipulative treatment was carried out with light touch to imitate chiropractic manipulative treatments (Sanders et al., 1990). The other study used a sham manipulation with light physical contact instead of professional kinesiology practice (PKP) (Eardley et al., 2013). In the last study, a noneffective force on a patient's hip and spine was used as a sham. This study also incorporated an enhanced sham group that received a suggestion that participants could expect pain relief alongside the sham manipulation (Bialosky et al., 2014).

##### Sham injection

Two of 18 studies utilized a sham injection to induce placebo effects (Charron et al., 2006; Pach et al., 2011). In one experimental study, placebo effects were assessed with a saline injection along with the verbal information that it was a potent analgesic with rapid effectiveness and compared to a saline injection with the information that it was an inert treatment (Charron et al., 2006). In the other study, participants in the placebo group received multiple injections of saline as a comparator to a subcutaneous injection with Disci/Rhus Toxicodendron Compositum, an anthroposophic drug used to treat acute LBP (Pach et al., 2011).

##### Sham oral medication

Sham oral medication was administered in 3 out of 18 studies (Carvalho et al., 2016; Ikemoto et al., 2020; Klinger et al., 2017). In 2 studies, sham pills were used to investigate the effect of open‐label placebo's (Carvalho et al., 2016; Ikemoto et al., 2020). The pills were administered along with the instruction that they were inert yet could lead to functional improvement as a consequence of placebo effects. In the third study, participants in the placebo group received a sham solution to mimic the effect of opioids. The placebo effect in this study was strengthened by a conditioning paradigm in half of the participants (Klinger et al., 2017).

##### Other placebo interventions

The remaining 6 out of 18 studies utilized different sham interventions: a temperature feedback sham intervention (Bush et al., 1985), a sham laser intervention (Borges et al., 2014), sham surgical tape (Pires et al., 2020) or sham ultrasound (Degenhardt et al., 2014; Faas et al., 1993).

##### Control groups

The control groups differed in the included studies (Table 1). In total, 5 out of 18 included studies had a no‐treatment control group that did not receive any intervention during the study (Bialosky et al., 2014; Borges et al., 2014; Degenhardt et al., 2014; Pires et al., 2020; Sanders et al., 1990). Another 6 studies assigned participants to a control group that received usual care (Carvalho et al., 2016; Cherkin et al., 2009; Faas et al., 1993; Ikemoto et al., 2020; Kim et al., 2020; Vas et al., 2012), which consisted of explanation of the symptoms and prescription of analgesic medication. Two studies assigned participants to a waiting list control group that received therapy after the waiting period (Bush et al., 1985; Eardley et al., 2013). Another 2 studies allowed participants allocated to the control group to use painkillers as back‐up medication (Leibing et al., 2002; Pach et al., 2011). In one study, the control group consisted of a waiting period, but participants were allowed to use back‐up medication during this period in case of severe pain (Brinkhaus et al., 2006). In two experimental placebo studies, participants in the control group received a control instruction, which stated that the therapy was/would be noneffective (Charron et al., 2006; Klinger et al., 2017).

#### 3.2.3 Outcome measures

Clinical pain intensity of LBP was assessed in 16 out of 18 studies, with 10 of them utilizing an NRS and the remaining 6 a VAS (Table 1). In 4 studies, pain intensity assessment was part of a pain questionnaire (Bialosky et al., 2014; Brinkhaus et al., 2006; Bush et al., 1985; Faas et al., 1993). The questionnaires used were the Daily Low Back Pain Record, McGill Pain Questionnaire, Pain Centered Outcomes Questionnaire, Nottingham Health Profile Questionnaire and a modified version of the German Pain Questionnaire or ‘Deutsche Schermzfragebogen’.

Experimental pain intensity of LBP was studied by 4 out of 18 studies. Two of these used quantitative sensory testing with the following subtests: pain pressure threshold, mechanical detection threshold, heat pain threshold, dynamic mechanical allodynia, heat temporal summation and aftersensation (Bialosky et al., 2014; Degenhardt et al., 2014). The remaining 2 studies measured pain intensity on an NRS after the application of electrical stimuli or a cold pressor test (Charron et al., 2006; Klinger et al., 2017).

LBP‐related disability was examined in 12 out of 18 studies and in 11 of them disability was quantified with questionnaires. The Roland Morris Disability Questionnaire was used in 5 studies (Carvalho et al., 2016; Cherkin et al., 2009; Eardley et al., 2013; Ikemoto et al., 2020; Vas et al., 2012). The Pain Disability Index was utilized in 3 studies (Brinkhaus et al., 2006; Leibing et al., 2002; Pach et al., 2011). In 2 German studies the Hannover Functional Ability Questionnaire was used (Klinger et al., 2017; Pach et al., 2011). The Oswestry Disability Index was utilized in one study (Bialosky et al., 2014). The final study made use of an NRS to measure LBP‐related disability (Faas et al., 1993).

#### 3.2.4 Placebo effects in clinical treatment studies investigating acute low back pain

##### Pain intensity

From the 3 studies investigating participants with acute LBP, only 1 reported a between‐group analysis for pain intensity and discovered no significant treatment effect of placebo (sham manipulation) over no‐treatment (Table 1) (Sanders et al., 1990). Of the remaining 2 studies, one study reported raw outcome data, and our testing of between‐group differences did not yield significant differences (*p* = 1.00) between placebo (sham ultrasound) and no‐treatment with an effect size of *SMD* = 0.00, 95% CI −0.23 to 0.23 (Faas et al., 1993). The third study did not report follow‐up data for pain intensity (Vas et al., 2012).

##### Disability

LBP‐related disability was examined by two studies that reported only raw outcome data (Faas et al., 1993; Vas et al., 2012). In one study, our testing of between‐group differences yielded a significant difference (*p* < .001) in disability improvement scores (RMDQ) favouring minimal acupuncture over no‐treatment with an effect size of *SMD* = 0.75, 95% CI 0.36 to 1.14 (Vas et al., 2012). In the other study, our testing of between‐group differences for the placebo (sham ultrasound) and no‐treatment groups was not significant (*p* = .65) with an effect size of *SMD* = −0.05, 95% CI −0.29 to 0.18 (Faas et al., 1993).

#### 3.2.5 Placebo effects in clinical treatment studies investigating subacute low back pain

The only study investigating participants with subacute LBP also included participants with chronic LBP and did not execute subgroup analyses (Bialosky et al., 2014). Therefore, specific placebo effects for participants with subacute LBP could not be studied.

#### 3.2.6 Placebo effects in clinical treatment studies investigating chronic low back pain

##### Pain intensity

From the 10 clinical studies investigating participants with chronic LBP (Borges et al., 2014; Brinkhaus et al., 2006; Bush et al., 1985; Cherkin et al., 2009; Degenhardt et al., 2014; Eardley et al., 2013; Kim et al., 2020; Leibing et al., 2002; Pach et al., 2011; Pires et al., 2020), a total of 9 studies investigated pain intensity and 5 reported a between‐group analysis. The analyses of all 5 studies showed no significant differences between the placebo (sham ultrasound, sham manipulation and sham exercise) and no‐treatment groups (Bush et al., 1985; Degenhardt et al., 2014; Eardley et al., 2013; Kim et al., 2020; Pires et al., 2020). However, one of these studies reported raw data that were incongruent with the outcomes of their between‐group analysis, and between‐group testing by our research group yielded a significant difference (*p* < .002) favouring sham manipulation over no‐treatment with an effect size of *SMD* = 1.09, 95% CI 0.40 to 1.78 (Eardley et al., 2013). The remaining 4 studies showed raw data for the placebo and no‐treatment groups and our testing for between‐group differences yielded two studies with significant differences (*p* < .01) favouring minimal acupuncture over no‐treatment with an effect size of *SMD* = 0.54, 95% CI 0.21 to 0.87 and *SMD =* 0.54, 95% CI 0.12 to 0.95 (Brinkhaus et al., 2006; Leibing et al., 2002). The data from the two remaining studies did not show significant differences between the placebo (sham ultrasound or sham injection) and no‐treatment groups (Borges et al., 2014; Pach et al., 2011). Overall, a total of 3 out of 9 studies reported a significant difference in pain intensity between groups, favouring sham manipulation and simulated acupuncture over no‐treatment.

##### Disability

LBP‐related disability was examined in 5 studies (Brinkhaus et al., 2006; Cherkin et al., 2009; Eardley et al., 2013; Leibing et al., 2002; Pach et al., 2011), of which 2 reported a between‐group analysis and discovered a significant effect favouring placebo (simulated acupuncture or sham manipulation) over no‐treatment (*p* < .05) (Cherkin et al., 2009; Eardley et al., 2013). The remaining 3 studies reported raw data for both groups and our examining of between‐group differences yielded two studies with significant differences (*p* = .02 and *p* < .001) favouring minimal acupuncture over no‐treatment with an effect size of *SMD* = 0.49, 95% CI 0.08 to 0.91 and *SMD =* 0.69, 95% CI 0.35 to 1.03 (Brinkhaus et al., 2006; Leibing et al., 2002). The raw data from the fifth study did not indicate significant differences between sham injection or no‐treatment groups (Pach et al., 2011). Overall, a total of 4 out of 5 studies reported a significant difference in disability between groups, favouring sham manipulation and simulated acupuncture over no‐treatment.

##### Pain bothersomeness

The influence of placebo effects on pain bothersomeness was analysed ad hoc as an addition to the evidence for pain‐related outcomes. A total of 3 studies investigating placebo effects in chronic LBP reported pain bothersomeness as an outcome (Carvalho et al., 2016; Cherkin et al., 2009; Kim et al., 2020). One study discovered a significant effect favouring placebo (simulated acupuncture) over no‐treatment (*p* < .05) with an effect size of *MD* = −1.56, 95% CI −2.11 to −1.02 (Cherkin et al., 2009). Two studies did not discover a significant difference between open‐label placebo pills and no‐treatment groups (*p* = .195 and *p* = .86) (Carvalho et al., 2016; Kim et al., 2020).

#### 3.2.7 Placebo effects in experimental studies

Four out of 18 studies specifically assessed placebo effects in an experimental setting (Carvalho et al., 2016; Charron et al., 2006; Ikemoto et al., 2020; Klinger et al., 2017). All four studies investigated participants with chronic LBP. The placebo treatments used were: an open label placebo pill twice a day for three weeks (Carvalho et al., 2016) or twelve weeks (Ikemoto et al., 2020), two sham injections with or without instruction on two different days (Charron et al., 2006), or a placebo instruction about an oral solution with or without a conditioning paradigm with experimental pain on one day (Klinger et al., 2017).

##### Pain intensity

All 4 studies executed between‐group analyses for pain intensity and all but one (Ikemoto et al., 2020) discovered statistically significant differences favouring placebo over no‐treatment. Two studies reported a significant effect size of *g* = 0.76 and *MD* = −2.83 (Carvalho et al., 2016; Klinger et al., 2017), whereas in the last one the effect size was not reported, nor were raw data available for analysis (Charron et al., 2006).

##### Disability

Three studies investigated LBP‐related disability (Carvalho et al., 2016; Ikemoto et al., 2020; Klinger et al., 2017) and 2 of them executed a between group analysis. One study showed a significant effect (*p* <.001) favouring placebo over no‐treatment with an effect size of *g* = 0.74 (Carvalho et al., 2016). The other study did not report a significant effect (*p* = .40) (Ikemoto et al., 2020). The third study investigating LBP‐related disability showed raw data for the placebo and no‐treatment groups. Our testing for between‐group differences yielded a significant difference (*p* = .01) favouring placebo over no‐treatment with an effect size of *SMD* = 1.12, 95% CI 0.24 to 1.99 (Klinger et al., 2017).

#### 3.2.8 Risk of bias (RoB) assessment

**FIGURE 2:**
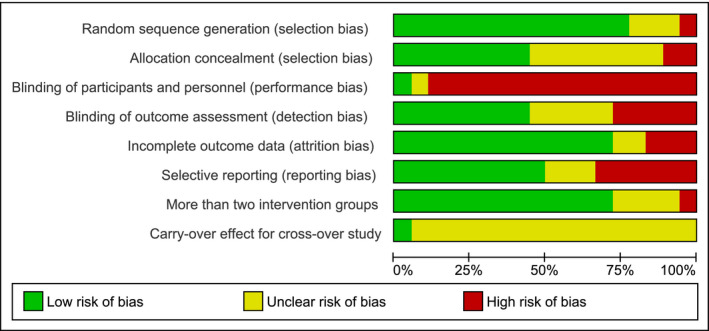
Risk of bias graph: review authors' judgments about each risk of bias item presented as percentages across all included studies. Performance bias was overall high (89%) as a result of the difficulties in blinding no‐treatment groups. The item ‘carry‐over effect for cross‐over studies' was scored frequently (94%) as ‘unclear risk’ due to the low amount of cross‐over trials (k = 1)

**FIGURE 3:**
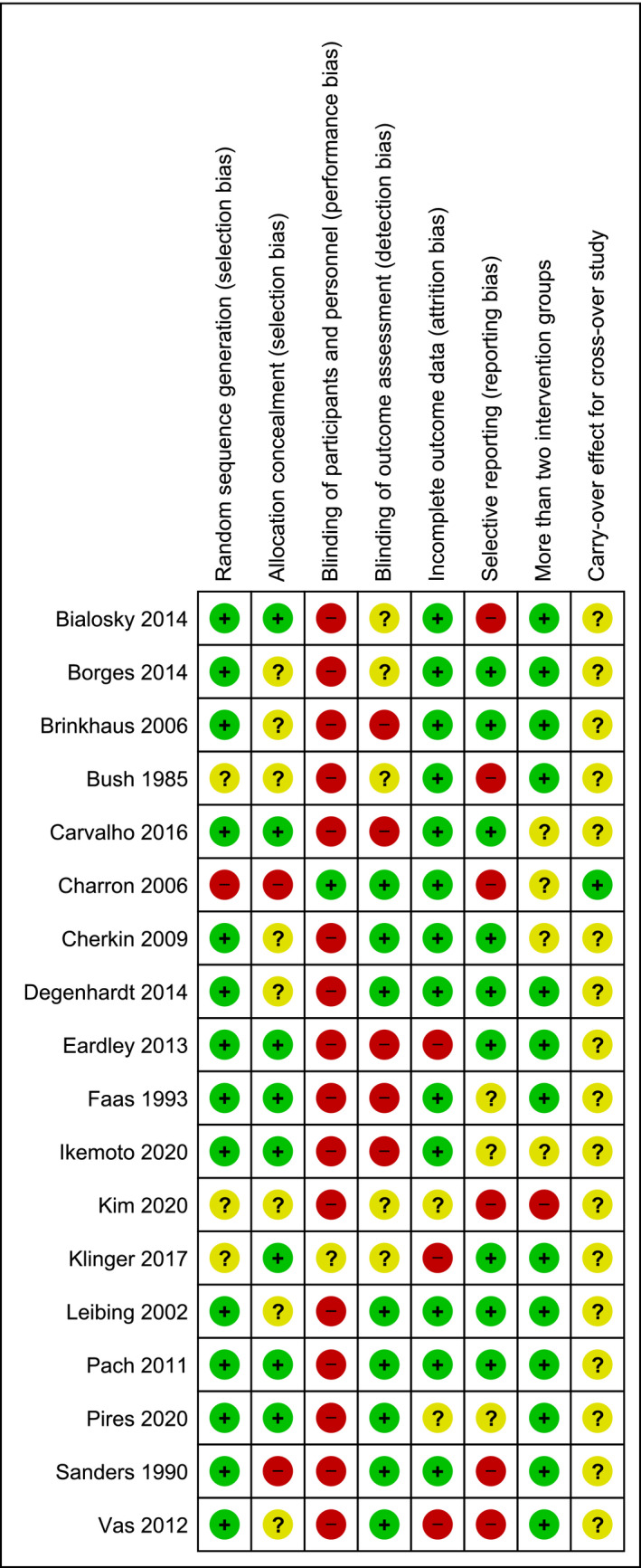
Risk of bias summary: review authors' judgments about each risk of bias item for each included study. Performance bias was overall high (89%) as a result of the difficulties in blinding no‐treatment groups. The item ‘carry‐over effect for cross‐over studies' was scored frequently (94%) as ‘unclear risk’ due to the low amount of cross‐over trials (k = 1)

The results of the RoB assessment are presented in Figures 2 and 3, showing the total amount of bias of all included studies per domain (Figure 2) and the bias results per individual study (Figure 3). Regarding the selection bias, 14 of the 18 studies had an adequate description of their randomization strategy. Of the remaining 4 studies, one study (Bush et al., 1985) mentioned stratification but not the exact method, another study (Charron et al., 2006) made use of pseudo‐randomization by nurses, and the remaining 2 studies did not specify the randomization technique (Kim et al., 2020; Klinger et al., 2017). Adequate concealment description was achieved in 8 studies. Two studies did not report allocation concealment, but their randomization method prevented adequate concealment and were considered high RoB (Charron et al., 2006; Sanders et al., 1990). The remaining 8 studies failed to adequately describe their allocation concealment and were considered unclear RoB (Borges et al., 2014; Brinkhaus et al., 2006; Bush et al., 1985; Cherkin et al., 2009; Degenhardt et al., 2014; Kim et al., 2020; Leibing et al., 2002; Vas et al., 2012). Performance bias was high in 16 studies due to the natural history or waiting list groups for which participants or personnel could not be adequately blinded. Since a participant does not receive any intervention in these groups, the participant and/or research personnel will be informed indirectly about the group allocation (Hróbjartsson, 2002). Two studies were not considered high RoB, since they specifically assessed placebo effects and contained a fake placebo rather than a natural history or waiting list group (Charron et al., 2006; Klinger et al., 2017). Blinding of outcome assessors was adequately described in 8 articles. Five studies did not describe this and were considered unclear RoB (Bialosky et al., 2014; Borges et al., 2014; Bush et al., 1985; Kim et al., 2020; Klinger et al., 2017). Four studies used patient‐reported outcomes when inadequately blinding patients, thereby creating high RoB (Brinkhaus et al., 2006; Carvalho et al., 2016; Eardley et al., 2013; Faas et al., 1993). In the last study, outcomes were assessed by the primary experimenter who also executed the intervention (Ikemoto et al., 2020). Three studies were at high risk for attrition bias, whereof two used a per‐protocol analysis (Eardley et al., 2013; Klinger et al., 2017), and one reported high drop‐out rates without a clarified description (Vas et al., 2012). In two studies there was an unclear risk of attrition bias due to relatively high drop‐out rates (Kim et al., 2020) and the absence of *P‐*level correction for multiple comparisons (Pires et al., 2020). One study also appeared to report an incorrect between‐group analysis for placebo and no‐treatment groups, based on the raw data presented in the paper (Eardley et al., 2013). Reporting bias was considered high in 6 studies, as they failed to depict raw data for pain outcomes (Bialosky et al., 2014; Bush et al., 1985; Charron et al., 2006; Kim et al., 2020; Sanders et al., 1990; Vas et al., 2012). All but one study used more than two intervention groups, but one of them created bias by pooling intervention groups to obtain significant results (Kim et al., 2020). The studies that did not compare more than two groups were scored ‘unclear’ because this aspect of bias assessment was not applicable (Carvalho et al., 2016; Charron et al., 2006; Cherkin et al., 2009; Ikemoto et al., 2020). One study used a cross‐over design and reported a carry‐over effect between different orders. However, because an adjusted analysis was performed, reporting bias was considered low (Charron et al., 2006).

#### 3.2.9 Meta‐analysis

**FIGURE 4:**
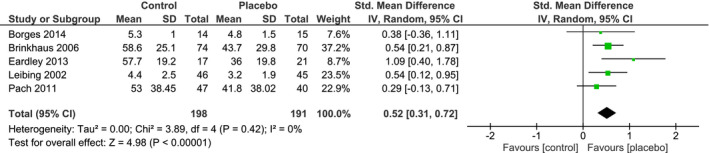
Forest plot of the random‐effects meta‐analysis showing the amount of pain relief (analgesia) in placebo groups versus no‐treatment groups in clinical treatment studies. Positive values for the Standardized Mean Difference indicate lower post‐intervention pain ratings in the placebo group than in the no‐treatment group

**FIGURE 5:**
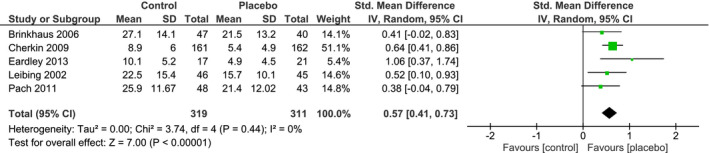
Forest plot of the random‐effects meta‐analysis showing the amount of disability improvement in placebo groups versus no‐treatment groups in clinical treatment studies. Positive values for the Standardized Mean Difference indicate lower post‐intervention pain ratings in the placebo group than in the no‐treatment group

From all the studies included in our review, only studies investigating chronic LBP were suitable for a meta‐analysis. Individual and pooled effect sizes are shown in Figures 4 and 5. The random effects meta‐analysis indicated a medium overall effect size favouring placebo over no‐treatment on LBP intensity (*SMD_pain_* = 0.52, 95% CI 0.31 to 0.72) and on LBP‐related disability (*SMD_disability_* = 0.57, 95% CI 0.41 to 0.73). The overall heterogeneity observed for LBP intensity and related disability was low (*I^2^* = 0%, τ^2^ = 0%). The RoB in the five studies assessing LBP intensity was overall low, only one study had a moderate RoB (Eardley et al., 2013). The RoB for the LBP‐related disability studies was overall low as well.

##### Sensitivity analysis

**FIGURE 6:**
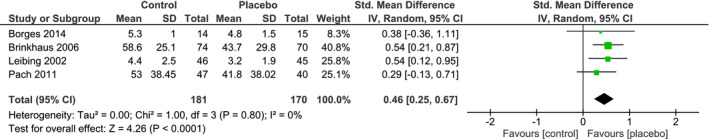
Forest plot of the sensitivity analysis showing the amount of pain relief (analgesia) in placebo groups versus no‐treatment groups in clinical treatment studies. One study was excluded from the sensitivity analysis due to the high RoB

**FIGURE 7:**
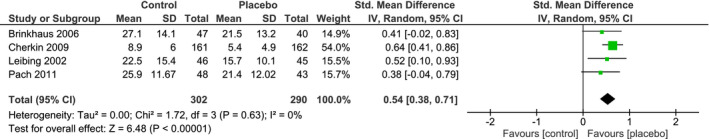
Forest plot of the sensitivity analysis showing the amount of disability improvement in placebo groups versus no‐treatment groups in clinical treatment studies. One study was excluded from the sensitivity analysis due to the high RoB

Visual inspection of box plots (Figure S1 and S2) depicting the individual effect sizes (expressed by SMDs) of LBP intensity and related disability yielded one study that could be considered a positive outlier (Eardley et al., 2013). This study also had a higher (moderate) RoB than all other included studies (low). Analysing the pooled effect estimates after excluding this study obtained somewhat lower but still statistically significant pooled effect sizes for LBP intensity and related disability (*SMD*_pain_ = 0.46, 95% CI = 0.25 to 0.67, *SMD*_disability_ = 0.54, 95% CI 0.38 to 0.71). Data for the sensitivity analysis are shown in Figures 6 and 7. Reporting bias was not assessed with funnel plots or an Eggers test due to the low number of trials that was included in the meta‐analysis.

## 4 DISCUSSION

Our systematic review examined the influence of placebo effects in patients with LBP by studying clinical treatment studies with placebo and no‐treatment groups, and experimental studies assessing placebo effects. The literature search yielded 18 clinical and experimental studies that were eligible for review. Clinical treatment studies were assessed according to LBP symptom duration (acute, subacute, or chronic). Three studies investigating patients with acute LBP indicated no major differences in LBP intensity and related disability between groups. A pooled effect size for placebo effects in acute LBP was not calculated due to incomplete outcome data in the included studies. There was an insufficient number of studies to draw any conclusions about the influence of placebo effects in patients with subacute LBP. Ten clinical treatment studies that investigated patients with chronic LBP were eligible for the systematic review. In the review, some evidence of placebo effects in LBP intensity was discovered with 3 out of 9 studies favouring placebo over no‐treatment groups. However, in LBP‐related disability the evidence was more prominent, with 4 out of 5 eligible studies depicting a statistically significant benefit of placebo over no‐treatment groups. An additional ad‐hoc analysis on placebo effects in LBP pain bothersomeness did not provide convincing evidence for placebo effects on this pain dimension in chronic LBP, as only 1 out of 3 studies showed a statistically significant difference favouring placebo over no‐treatment groups. The 4 experimental studies showed substantial evidence for placebo effects in chronic LBP. In the meta‐analysis, a significant moderate effect of placebo interventions over no‐treatment controls for both LBP intensity and related disability was discovered. Even after exclusion of one study that had the highest RoB and was a positive outlier, the effect size of the placebo interventions remained similar.

The results of our review seem to indicate that placebo effects have a more prominent role in chronic LBP then in (sub)acute LBP, although the number of studies in acute and subacute LBP was minimal. The findings on chronic LBP corroborate earlier evidence that chronic pain patients react differently to placebo effects due to altered psychological components of pain processing, including negative emotions and cognitions, and an increased susceptibility to internal predictions (Kaptchuk et al., 2020; Skyt et al., 2020). Additional studies investigating the influence of these psychological components on susceptibility for placebo effects could lead to a more patient‐focused and successful implementation of placebo treatments in clinical practice (Kaptchuk et al., 2020).

The results of our review regarding the presence of placebo effects in LBP further add to the previous best‐evidence synthesis by Puhl et al., and emphasize the importance of placebo effects due to contextual factors in chronic LBP treatment (Puhl et al., 2011; Testa & Rossettini, 2016). The low to moderate effect sizes seen in LBP treatments might even be predominantly caused by placebo effects, as is suggested by Stilwell and Harman in the case of exercise treatments (Stilwell & Harman, 2017). According to the authors different exercise treatments seem to have similar effect sizes, possibly due to common contextual factors that create placebo effects (Stilwell & Harman, 2017). The moderate size of the placebo effect found in our review provides further evidence for this theory and stresses the use of contextual factors in treating patients with chronic LBP. These factors could for instance be adopted in a biopsychosocial treatment approach, which has already proven to be more effective than usual care for LBP (Kamper et al., 2014).

Next to the size of the placebo effect, different sham interventions were also assessed in this review. The results revealed that sham acupuncture (minimal or simulated) and sham manipulation led to significant placebo effects. A finding that was not in line with the previous review (Puhl et al., 2011), which concluded that sham laser and sham medication caused clinically meaningful improvements. One explanation for the difference in findings is that in the current review placebo effects were investigated by comparing placebo with no‐treatment groups instead of looking at placebo groups alone, as Puhl et al. did. This led to the inclusion of different trials in both reviews. The predominant reasons, mentioned by Puhl et al., for not investigating trials with no‐treatment groups, are the ethical concern of withholding patients from medical treatment and the small number of trials that contain no‐treatment groups (Puhl et al., 2011). Interestingly, the authors did not discuss studies comparing placebo to waiting list groups or comparing placebo (with usual care) to usual care alone. Including these studies in a systematic review increases the amount of available data and provides essential evidence for the role of placebo effects in LBP. Although studies that incorporate usual care groups might lead to co‐intervention bias, as a result of substantially increased medication use. The use of medication can be treated as a covariate and an appropriate analysis that corrects for this covariate could then reveal true placebo effects. Another explanation for the difference in findings is the questioned legitimacy of some placebo treatments (e.g., minimal acupuncture) (Puhl et al., 2011). During these placebo treatments, LBP patients experience physical pain, which might activate descending pain pathways resulting in a neurophysiological effect similar to active medical treatments (Lundeberg et al., 2011). According to the authors these placebo treatments can therefore not be deemed an adequate placebo intervention (Puhl et al., 2011). Although this reasoning might seem logical, studies that investigated the effect of direct noxious stimuli on pain inhibition by descending pathways (conditional pain modulation) in LBP showed no evidence of this (Correa et al., 2015). Apart from the physical pain experience, some researchers note that behavioural conditioning, an essential psychological learning mechanism behind placebo effects (Babel, 2019; Pavlov, 1928), is a crucial element in some medical treatments and part of true treatment effects (Lundeberg et al., 2011). However, one might then argue what the biomedical influence of these treatments is and whether their efficacy is not primarily due to placebo effects (Song et al., 2017). This statement is in line with our previous conclusion about the importance of contextual factors around LBP treatment and raises the question whether placebo treatments could be adopted as legitimate treatment options or as add‐ons to existing treatment modalities for patients with LBP in clinical practice (Benedetti et al., 2003). For now, this review points to minimal acupuncture and sham manipulation as potential placebo treatments to result in pain intensity and disability improvements in patients with LBP.

There are several limitations of this systematic review that should be addressed. The number of studies included in the review is relatively low. This is mainly due to the comparison of placebo interventions versus no‐treatment control groups, as most treatment trials either incorporate a placebo arm to test effectiveness or a usual care arm for pragmatic reasons (Goldstein et al., 2018). As a result of the low amount of trials, a limited sensitivity analysis was conducted and a subgroup analysis was lacking, restricting conclusions about possible reporting bias or different kinds of placebo interventions (Higgins & Green, 2011). Other limitations are possible performance bias and co‐intervention bias occurring in the no‐treatment control groups. The no‐treatment control groups create performance bias due to the lack of blinding (Hróbjartsson, 2002). This also creates the possibility that, although both groups were allowed routine care, participants in the no‐treatment groups received different care compared with participants in the placebo groups (co‐intervention bias) (Hróbjartsson, 2002). Finally, both outcomes, LBP intensity and LBP‐related disability are patient‐reported, which makes them susceptible for response bias, especially in case of the nonblinded no‐treatment groups. It is important to note, though, that response bias and co‐intervention bias might partly cancel each other out instead of amplifying one another (Hrobjartsson & Gotzsche, 2010).

Several future directions based on this review can be considered for further examination of the role of placebo effects in LBP. Our meta‐analysis demonstrated a moderate effect of placebo treatments in chronic LBP intensity or related disability between 5 and 12 weeks, but more extensive follow‐up studies of several months to years are warranted to assess long‐term benefit (Krismer & van Tulder, 2007). Furthermore, additional RCTs investigating LBP with placebo and usual care arms could provide a possibility for more extensive meta‐analysis and a more reliable estimate of the effect size. Lastly, the amount of experimental trials assessing placebo effects in LBP, although growing, is still limited. This restricts the conclusions that can be drawn about the effectiveness of placebo effects and possible placebo treatments in LBP (Forsberg et al., 2017).

## 5 CONCLUSION

Our meta‐analysis provides evidence for a moderate influence of placebo effects in chronic LBP intensity as well as related disability. The number of trials investigating acute and subacute LBP were insufficient to draw any conclusions about the pooled effect size of placebo interventions. Nonetheless, individual effect sizes of these studies were overall medium to high. The conclusion that placebo effects significantly influence chronic LBP further emphasizes the importance of contextual factors around regular LBP treatments.

## CONFLICT OF INTEREST

The authors have no conflicts of interest to declare.

## AUTHOR CONTRIBUTIONS

J.P.A. van Lennep drafted the protocol, reviewed citations and full texts for eligibility, extracted data from full text articles, performed risk of bias assessment and statistical analyses, drafted the manuscript. F. Trossèl reviewed citations and full texts for eligibility, performed risk of bias assessments, and drafted the manuscript. R.S.G.M. Perez initiated the review project, drafted the protocol with J.P.A. van Lennep, passed away during the search stage. R.H.J. Otten developed the search strategy and executed the search with J.P.A. van Lennep. H. van Middendorp contributed to the manuscript. A.W.M. Evers contributed to the manuscript. K.M. Szadek became projectleader after R.S.G.M. Perez passed away, contributed to the protocol, reviewed citations and full texts for eligibility, reviewed risk of bias assessments, contributed to the manuscript. All authors reviewed and discussed the results, and commented on the manuscript.

## Supporting information

Fig S1

Fig S2

Appendix S1
