# Supplementary figures and images for: Placebo effects in low back pain: A systematic review and meta‐analysis of the literature
Source: Eur J Pain. 2021 Jun 21;25(9):1876–97. doi: 10.1002/ejp.1811 (PMC8518410; doi:10.1002/ejp.1811)

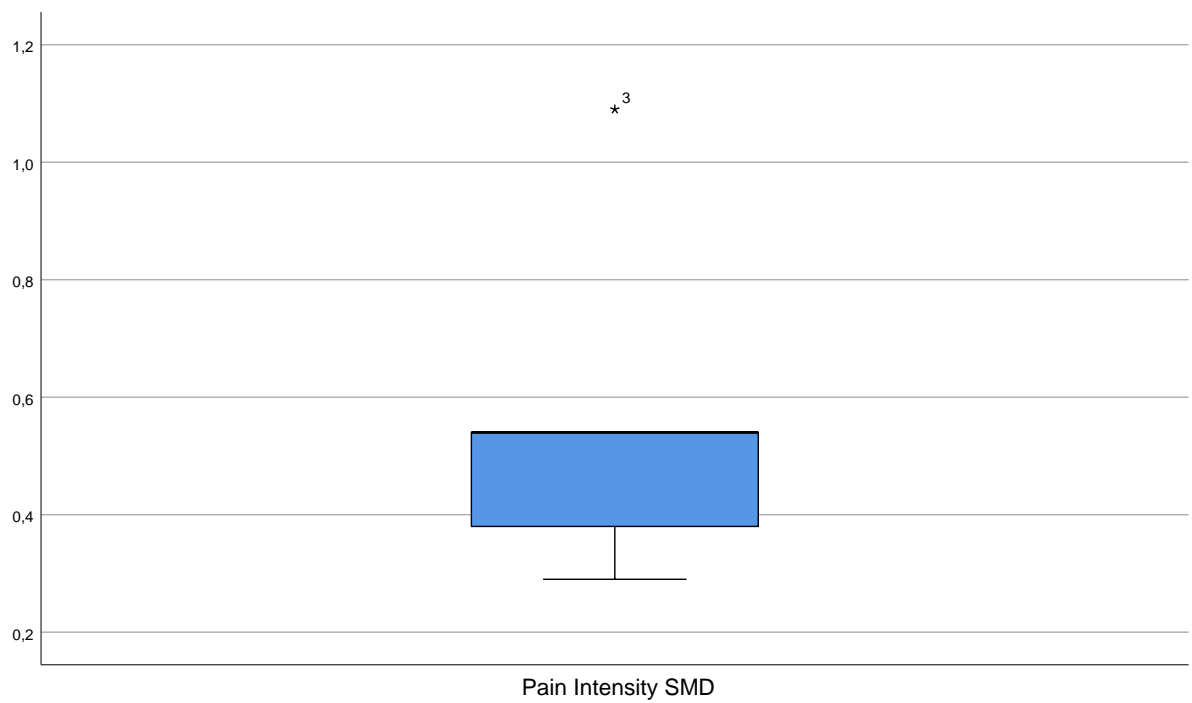

Supplement: Supplementary file 1 — Fig S1 [file EJP-25-1876-s001.pdf]

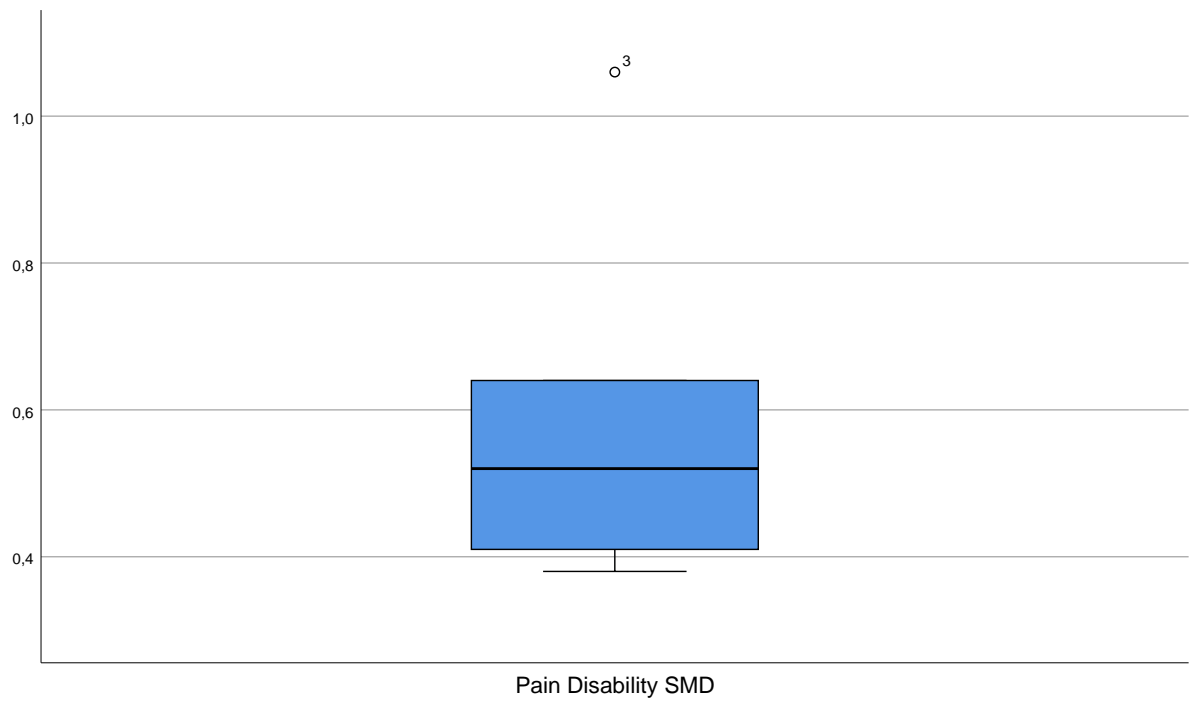

Supplement: Supplementary file 2 — Fig S2 [file EJP-25-1876-s003.pdf]
