# Supplementary material for: Placebo effects in low back pain: A systematic review and meta‐analysis of the literature
Source: Eur J Pain. 2021 Jun 21;25(9):1876–97. doi: 10.1002/ejp.1811 (PMC8518410; doi:10.1002/ejp.1811)
Supplement: Supplementary file 3 — Appendix S1 [file EJP-25-1876-s002.docx]

**PubMed**

"Low Back Pain"[Mesh] OR "Back Pain"[Mesh]

back ache*[tiab] OR back dysfunction*[tiab] OR back pain*[tiab] OR back strain*[tiab] OR backache*[tiab] OR loin pain*[tiab] OR low back ache*[tiab] OR low back pain*[tiab] OR low back syndrome*[tiab] OR low backache*[tiab] OR low backpain*[tiab] OR lowback pain*[tiab] OR “lower back ache”[tiab] OR “lower back aches”[tiab] OR lower back pain*[tiab] OR “lower back syndrome”[tiab] OR “lower back syndromes”[tiab] OR lower backache*[tiab] OR lower backpain*[tiab] OR lumbago*[tiab] OR lumbal pain*[tiab] OR lumbal syndrome*[tiab] OR lumbalgesi*[tiab] OR lumbalgia*[tiab] OR lumbar pain*[tiab] OR lumbar spine syndrome*[tiab] OR lumbar syndrome*[tiab] OR lumbodynia*[tiab] OR lumbosacral pain*[tiab] OR “lumbosacral root syndrome”[tiab] OR “lumbosacral root syndromes”[tiab] OR “lumbosacroiliac strain”[tiab] OR “lumbosacroiliac strains”[tiab] OR muscle pain*[tiab] OR muscular pain*[tiab] OR musculoskeletal pain*[tiab] OR NSLBP[tiab] OR Sciatica*[tiab] OR vertebrogenic pain syndrome*[tiab]

"Placebo Effect"[Mesh:NoExp]

placebo effect*[tiab] OR placebo reaction*[tiab] OR placebo response*[tiab]

"Placebos"[Mesh] OR "Counterfeit Drugs"[Mesh]

Placebo*[tiab] OR Counterfeit*[tiab] OR Dummy[tiab] OR Fake*[tiab] OR Sham[tiab]

Placebo*[tiab] OR Counterfeit drug*[tiab] OR Counterfeit medicine*[tiab] OR Dummy medical treatment*[tiab] OR Dummy medication*[tiab] OR Dummy treatment*[tiab] OR Fake drug*[tiab] OR Fake medicine*[tiab] OR Sham operation*[tiab] OR Sham procedure*[tiab] OR Sham therap*[tiab] OR Sham treatment*[tiab]

**EMBASE**

'low back pain'/exp OR 'backache'/exp

‘back ache*’:ti,ab OR ‘back dysfunction*’:ti,ab OR ‘back pain*’:ti,ab OR ‘back strain*’:ti,ab OR ‘backache*’:ti,ab OR ‘backpain*’:ti,ab OR ‘loin pain*’:ti,ab OR ‘low back syndrome*’:ti,ab OR ‘low backache*’:ti,ab OR ‘low backpain*’:ti,ab OR ‘lowback pain*’:ti,ab OR ‘lowerback pain*’:ti,ab OR ‘lower back syndrome*’:ti,ab OR ‘lower backache*’:ti,ab OR ‘lower backpain*’:ti,ab OR ‘lumbago*’:ti,ab OR ‘lumbal pain*’:ti,ab OR ‘lumbal syndrome*’:ti,ab OR ‘lumbalgesi*’:ti,ab OR ‘lumbalgia*’:ti,ab OR ‘lumbar adjacent pain*’:ti,ab OR ‘lumbar pain*’:ti,ab OR ‘lumbar spine syndrome*’:ti,ab OR ‘lumbar syndrome*’:ti,ab OR ‘lumbodynia*’:ti,ab OR ‘lumbosacral pain*’:ti,ab OR ‘lumbosacral root syndrome*’:ti,ab OR ‘lumbosacroiliac strain*’:ti,ab OR ‘muscle pain*’:ti,ab OR ‘muscular pain*’:ti,ab OR ‘musculoskeletal pain*’:ti,ab OR ‘NSLBP’:ti,ab OR ‘Sciatica*’:ti,ab OR ‘vertebrogenic pain syndrome*’:ti,ab

'placebo effect'/exp

'placebo effect*':ti,ab OR 'placebo reaction*':ti,ab OR 'placebo response*':ti,ab

'placebo'/exp OR 'sham procedure'/exp OR 'counterfeit drug'/exp

Placebo*:ti,ab OR Counterfeit*:ti,ab OR Dummy:ti,ab OR Fake*:ti,ab OR Sham:ti,ab

‘Placebo*’:ti,ab OR ‘Counterfeit drug*’:ti,ab OR ‘Counterfeit medicine*’:ti,ab OR ‘Dummy medical treatment*’:ti,ab OR ‘Dummy medication*’:ti,ab OR ‘Dummy treatment*’:ti,ab OR ‘Fake drug*’:ti,ab OR ‘Fake medicine*’:ti,ab OR ‘Sham operation*’:ti,ab OR ‘Sham procedure*’:ti,ab OR ‘Sham therap*’:ti,ab OR ‘Sham treatment*’:ti,ab

**COCHRANE LIBRARY**

“back ache*”:ti,ab,kw OR “back dysfunction*”:ti,ab,kw OR “back pain*”:ti,ab,kw OR “back strain*”:ti,ab,kw OR “backache*”:ti,ab,kw OR “backpain*”:ti,ab,kw OR “loin pain*”:ti,ab,kw OR “low back syndrome*”:ti,ab,kw OR “low backache*”:ti,ab,kw OR “low backpain*”:ti,ab,kw OR “lowback pain*”:ti,ab,kw OR “lowerback pain*”:ti,ab,kw OR “lower back syndrome*”:ti,ab,kw OR “lower backache*”:ti,ab,kw OR “lower backpain*”:ti,ab,kw OR “lumbago*”:ti,ab,kw OR “lumbal pain*”:ti,ab,kw OR “lumbal syndrome*”:ti,ab,kw OR “lumbalgesi*”:ti,ab,kw OR “lumbalgia*”:ti,ab,kw OR “lumbar adjacent pain*”:ti,ab,kw OR “lumbar pain*”:ti,ab,kw OR “lumbar spine syndrome*”:ti,ab,kw OR “lumbar syndrome*”:ti,ab,kw OR “lumbodynia*”:ti,ab,kw OR “lumbosacral pain*”:ti,ab,kw OR “lumbosacral root syndrome*”:ti,ab,kw OR “lumbosacroiliac strain*”:ti,ab,kw OR “muscle pain*”:ti,ab,kw OR “muscular pain*”:ti,ab,kw OR “musculoskeletal pain*”:ti,ab,kw OR “NSLBP”:ti,ab,kw OR “Sciatica*”:ti,ab,kw OR “vertebrogenic pain syndrome*”:ti,ab,kw

Resultaat: 10271

“placebo effect*”:ti,ab,kw OR “placebo reaction*”:ti,ab,kw OR “placebo response*”:ti,ab,kw

Resultaat: 3500

#1 and #2 resultaat: 116

2 cochrane reviews

1 other review

107 trials

6 method studies

Placebo*:ti,ab,kw OR Counterfeit*:ti,ab,kw OR Dummy:ti,ab,kw OR Fake*:ti,ab,kw OR Sham:ti,ab,kw

**CINAHL**

(MH "Back Pain") OR (MH "Low Back Pain")

TI (“back ache*” OR “back dysfunction*” OR “back pain*” OR “back strain*” OR “backache*” OR “backpain*” OR “loin pain*” OR “low back syndrome*” OR “low backache*” OR “low backpain*” OR “lowback pain*” OR “lowerback pain*” OR “lower back syndrome*” OR “lower backache*” OR “lower backpain*” OR “lumbago*” OR “lumbal pain*” OR “lumbal syndrome*” OR “lumbalgesi*” OR “lumbalgia*” OR “lumbar adjacent pain*” OR “lumbar pain*” OR “lumbar spine syndrome*” OR “lumbar syndrome*” OR “lumbodynia*” OR “lumbosacral pain*” OR “lumbosacral root syndrome*” OR “lumbosacroiliac strain*” OR “muscle pain*” OR “muscular pain*” OR “musculoskeletal pain*” OR “NSLBP” OR “Sciatica*” OR “vertebrogenic pain syndrome*”) OR AB (“back ache*” OR “back dysfunction*” OR “back pain*” OR “back strain*” OR “backache*” OR “backpain*” OR “loin pain*” OR “low back syndrome*” OR “low backache*” OR “low backpain*” OR “lowback pain*” OR “lowerback pain*” OR “lower back syndrome*” OR “lower backache*” OR “lower backpain*” OR “lumbago*” OR “lumbal pain*” OR “lumbal syndrome*” OR “lumbalgesi*” OR “lumbalgia*” OR “lumbar adjacent pain*” OR “lumbar pain*” OR “lumbar spine syndrome*” OR “lumbar syndrome*” OR “lumbodynia*” OR “lumbosacral pain*” OR “lumbosacral root syndrome*” OR “lumbosacroiliac strain*” OR “muscle pain*” OR “muscular pain*” OR “musculoskeletal pain*” OR “NSLBP” OR “Sciatica*” OR “vertebrogenic pain syndrome*”)

(MH "Placebo Effect")

TI (“placebo effect*” OR “placebo reaction*” OR “placebo response*”) OR AB (“placebo effect*” OR “placebo reaction*” OR “placebo response*”)

(MH "Placebos") OR (MH "Counterfeit Drugs")

TI (Placebo* OR Counterfeit* OR Dummy OR Fake* OR Sham) OR AB (Placebo* OR Counterfeit* OR Dummy OR Fake* OR Sham)

**PsycINFO**

DE "Back Pain"

TI (“back ache*” OR “back dysfunction*” OR “back pain*” OR “back strain*” OR “backache*” OR “backpain*” OR “loin pain*” OR “low back syndrome*” OR “low backache*” OR “low backpain*” OR “lowback pain*” OR “lowerback pain*” OR “lower back syndrome*” OR “lower backache*” OR “lower backpain*” OR “lumbago*” OR “lumbal pain*” OR “lumbal syndrome*” OR “lumbalgesi*” OR “lumbalgia*” OR “lumbar adjacent pain*” OR “lumbar pain*” OR “lumbar spine syndrome*” OR “lumbar syndrome*” OR “lumbodynia*” OR “lumbosacral pain*” OR “lumbosacral root syndrome*” OR “lumbosacroiliac strain*” OR “muscle pain*” OR “muscular pain*” OR “musculoskeletal pain*” OR “NSLBP” OR “Sciatica*” OR “vertebrogenic pain syndrome*”) OR AB (“back ache*” OR “back dysfunction*” OR “back pain*” OR “back strain*” OR “backache*” OR “backpain*” OR “loin pain*” OR “low back syndrome*” OR “low backache*” OR “low backpain*” OR “lowback pain*” OR “lowerback pain*” OR “lower back syndrome*” OR “lower backache*” OR “lower backpain*” OR “lumbago*” OR “lumbal pain*” OR “lumbal syndrome*” OR “lumbalgesi*” OR “lumbalgia*” OR “lumbar adjacent pain*” OR “lumbar pain*” OR “lumbar spine syndrome*” OR “lumbar syndrome*” OR “lumbodynia*” OR “lumbosacral pain*” OR “lumbosacral root syndrome*” OR “lumbosacroiliac strain*” OR “muscle pain*” OR “muscular pain*” OR “musculoskeletal pain*” OR “NSLBP” OR “Sciatica*” OR “vertebrogenic pain syndrome*”)

TI (“placebo effect*” OR “placebo reaction*” OR “placebo response*”) OR AB (“placebo effect*” OR “placebo reaction*” OR “placebo response*”)

DE "Placebo"

TI (Placebo* OR Counterfeit* OR Dummy OR Fake* OR Sham) OR AB (Placebo* OR Counterfeit* OR Dummy OR Fake* OR Sham)

No care

No contact

No intervention

No medication

No operation

No operative

No surgery

No surgical

No therapies

No therapy

No treated

No treatment

No treatments

Non contact

Non intervention

Non medication

Non operation

Non operative

Non surgery

Non surgical

Non therapy

Non treated

Non treatment

Not operated

Not operative

Not treated

Nonintervention

Nonmedication

Nonoperated

Nonoperative

Nonsurgical

Nontherapeutical

Nontreated

nontreatment

minimal care

minimal contact

minimal intervention

minimal medication

minimal treatment

minimally treated

Untreated

Usual care

Usual therapy

Usual therapies

Usual treatment
